# Kaposi Sarcoma of the Ampulla of Vater and AIDS Cholangiopathy: A Rare Biliary Presentation

**DOI:** 10.14309/crj.0000000000002325

**Published:** 2026-10-30

**Authors:** Jayasree Rajapandian, Choudhary Ahmed Hasan Maqbool, Yousif Elmofti

**Affiliations:** 1 Heersink School of Medicine, Montgomery Regional Campus, University of Alabama at Birmingham, Montgomery, AL

**Keywords:** Kaposi sarcoma, AIDS cholangiopathy, ERCP

## Abstract

Biliary manifestations in advanced acquired immunodeficiency syndrome (AIDS) present unique diagnostic challenges. We present a case of Kaposi sarcoma of the ampulla of Vater with AIDS cholangiopathy in a 25-year-old man. He presented with watery nonbloody diarrhea and loss of weight for 2 months with a cholestatic liver pattern. Magnetic resonance cholangiopancreatography showed hepatic lesions possibly Kaposi sarcoma and irregular bile and pancreatic ducts with a suspected stricture at the ampulla consistent with AIDS cholangiopathy. Endoscopic retrograde cholangiopancreatography confirmed a vascular, umbilicated lesion in the ampulla of Vater, which proved biliary stenting difficult. This case highlights the diagnostic complexity of these rare presentations of HIV.

## INTRODUCTION

Kaposi sarcoma (KS) is a low-grade angioproliferative tumor of the vascular and lymphatic endothelium, most commonly seen in patients with HIV and CD4 counts below 100. While gastrointestinal tract involvement is the most frequent site of extracutaneous involvement, biliary tract disease remains exceedingly rare.^1^ The prevalence of acquired immunodeficiency syndrome (AIDS) cholangiopathy has also declined significantly in the highly active antiretroviral therapy (HAART) era and is now primarily encountered in patients with limited access to care or poor adherence to therapy. This case highlights the diagnostic and therapeutic challenges posed by the uncommon co-occurrence of KS of the ampulla of Vater and AIDS cholangiopathy, emphasizing both the technical difficulty of biliary intervention and the prognostic implications in advanced AIDS.

## CASE REPORT

A 25-year-old man with untreated HIV, complicated by cutaneous KS and oropharyngeal candidiasis, presented 2 months of nonbloody loose stools, weight loss, and decreased appetite. He appeared severely malnourished with a body mass index of 16. Physical examination showed mild right upper quadrant tenderness. Laboratory studies revealed worsening liver function tests, including alkaline phosphatase—1883 U/L, aspartate aminotransferase—235 U/L, alanine transaminase—198 U/L, and direct bilirubin—1.7 mg/dL. His CD4 count was 106, with an HIV RNA of 2024. Stool culture, including ova and parasite testing, was negative for cryptosporidium and giardia antigen. *Clostridioides difficile* testing with NAAT was negative as well. He was also found to have cytomegalovirus (CMV) viremia and was started on intravenous ganciclovir. Other fungal serologies and cryptosporidium antigen testing of the blood were revealed negative. Abdominal/pelvic CT showed around 10 small ill-defined hypoattenuating lesions in the liver suspicious of hepatic KS. Magnetic resonance cholangiopancreatography (MRCP) of the abdomen revealed a stricture ring at the ampulla with a mildly dilated common bile duct along with irregular bile and pancreatic ducts with few small outpouchings. These findings were consistent with type IV AIDS cholangiopathy. A hepatobiliary iminodiacetic acid scan confirmed cholestasis with a “liver-only” pattern, likely due to biliary papillary stenosis. Subsequent endoscopic retrograde cholangiopancreatography (ERCP) revealed a large 2–3 cm, bluish-red vascular umbilical mass at the ampulla, suspicious for KS. Biopsy with cold forceps confirmed the same, noting avascular proliferation with partially ectatic vessels and other areas with cellular spindling and extravasated blood vessels. Immunoperoxidase staining with human herpesvirus-8 was positive. The biliary orifice was identified, but cannulation of the CBD was unsuccessful despite multiple attempts given the ampullary mass. Therefore, cholangiography and biliary brushing were not performed. However, there was some bile flow noted at the end of the procedure. An endoscopic ultrasound (EUS)-guided choledochoduodenostomy was considered for biliary decompression, and plans were made to transfer the patient to a higher level of care for advanced endoscopic management. In the interim, although systemic therapy with liposomal doxorubicin was considered, significant concerns were raised regarding the patient's ability to tolerate treatment in the setting of advanced AIDS, disseminated KS, and progressive clinical decline, with additional concern for immune reconstitution inflammatory syndrome. Unfortunately, the patient's condition rapidly deteriorated, culminating in acute respiratory failure requiring intubation, and he subsequently expired before further therapeutic measures could be undertaken (Figure 1).

**Figure 1.**
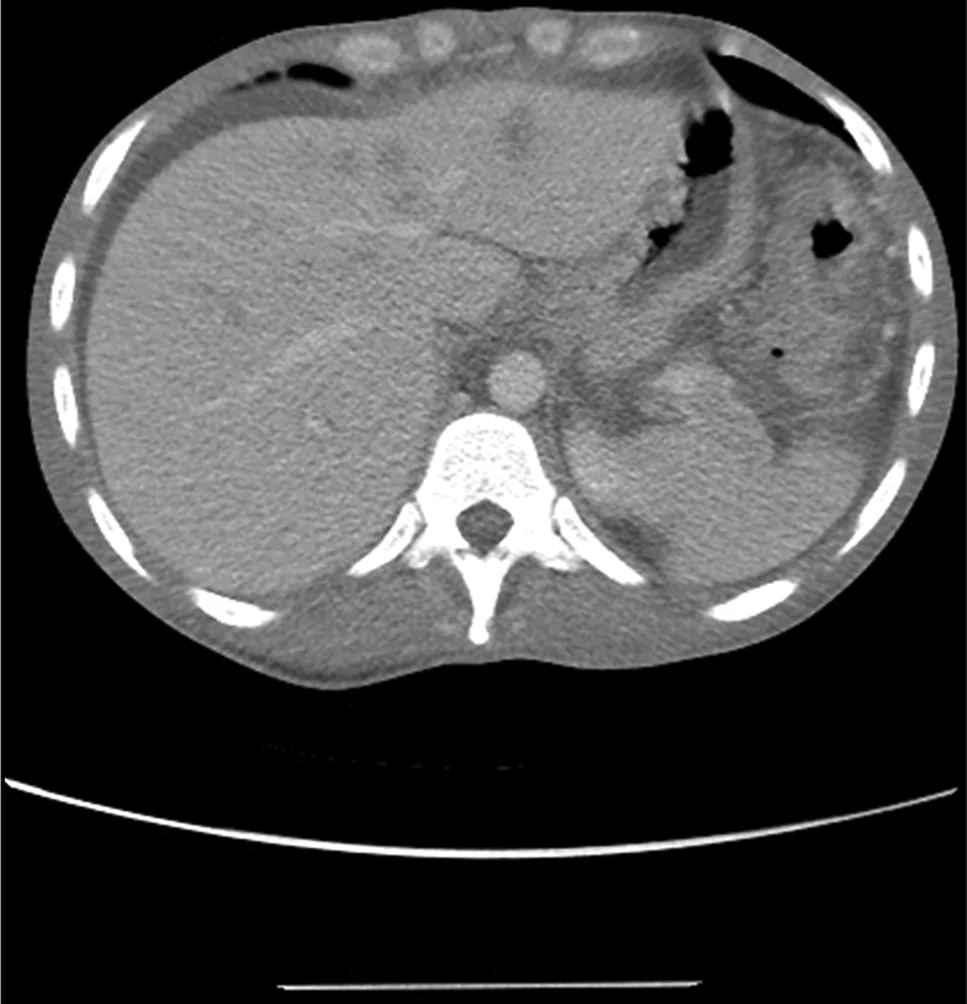
Abdominal/pelvic CT showing multiple ill-defined hypodense lesions in the liver suspicious of Kaposi sarcoma.

## DISCUSSION

Gastrointestinal and hepatobiliary involvement are well-recognized complications of advanced AIDS, most commonly resulting from opportunistic infections in the setting of severe immunosuppression. KS, caused by HHV-8 infection, is 1 such disease that presents in 4 clinical variants: classic (Mediterranean), endemic (African), epidemic (AIDS-related), and iatrogenic (post-transplant).^2^ AIDS-related KS of the gastrointestinal tract is seen in 80% of patients with cutaneous KS and indicates poor prognosis. It remains asymptomatic in most of the individuals, with only 1 in 5 patients experiencing symptoms, which range from vague upper abdominal pain, nausea, vomiting, and malabsorption to upper or lower gastrointestinal (GI) bleeding.^1^ Some authors even recommend routine endoscopic evaluation in patients with cutaneous KS for early detection and diagnosis, especially in those with CD4 counts <100 and men who have sex with men.^3,4^

Hepatic involvement with KS is seen in 12%–24% of patients with AIDS on incidental imaging and in 35% of the patients with cutaneous KS during autopsy. Biliary involvement of KS is rarer still.^5^ In a large SEER-based study of 685 primary gastrointestinal KS cases (1975–2019) published in 2022, luminal GI KS accounted for 84.96% of cases while solid organ involvement (liver, pancreas, and gallbladder) represented only 3.07% of all GI KS cases.^6^ In our patient, abdominal/pelvic CT with contrast showed numerous hypodense lesions in the liver, which were likely KS. Unfortunately, we were unable to biopsy the lesion before the patient deteriorated. Hepatic KS is typically asymptomatic. They usually appear as inhomogeneous hypodense nodules along the peripheral branches of portal veins as was seen in the CT. The combination of antiretroviral therapy and liposomal doxorubicin is an effective treatment strategy. In 1 study, this combination resulted in a 5-year overall survival rate of 85% in 140 patients with T1 disease.^7^ In our patient, however, the decision to initiate liposomal doxorubicin was complicated by his poor functional status and concern that risks might outweigh potential benefit.

While hepatobiliary involvement by KS is rare, AIDS cholangiopathy was seen in 26%-46% of patients in the pre-HAART era. It represents a form of infectious sclerosing cholangitis predominantly caused by CMV or *Cryptosporidium*, though *Microsporidium*, *Cyclospora*, and *Isospora* have also been implicated.^8^ Infection with *Cryptosporidium* is associated with worse outcomes. Pathogenesis involves ischemic injury from CMV and direct epithelial apoptosis and inflammation from *Cryptosporidium*. This causes 4 main patterns: papillary stenosis (type I, 20% of cases), sclerosing cholangitis (type II, 15%–20%), combined involvement (type III, 50%) and extrahepatic strictures with or without intrahepatic involvement (type IV, 6%–15%).^9,10^ The presence of irregular biliary and pancreatic ducts with multiple small ductal outpouchings, in conjunction with only mild common bile duct dilation (8 mm) in the MRCP, argues against obstruction at the ampulla of Vater by the KS lesion as the primary etiology. Rather, these characteristic cholangiographic findings are most consistent with AIDS cholangiopathy.

Existing literature on biliary involvement in advanced AIDS illustrates a spectrum of diseases that differ from our case. Previous reports have described AIDS cholangiopathy secondary to* Cryptosporidium* and CMV coinfection in the absence of any structural or neoplastic ampullary lesion, with poor outcomes despite antimicrobial therapy and resumption of HAART.^11^ Similarly, other reports have described the characteristic imaging findings of AIDS cholangiopathy in patients with cryptosporidiosis, again without an associated mass.^12^ In another published case, disseminated KS causing extrahepatic cholestasis from a discrete stricture of the common hepatic duct. ERCP with sphincterotomy and stenting was technically feasible because the lesion was not located at the ampulla. This intervention had produced marked biochemical improvement, although the patient's overall course remained limited by profound underlying immunosuppression.^13^ By contrast, our patient had a KS lesion specifically at the ampulla of Vater, an anatomic location that precluded biliary cannulation altogether. This unique presentation distinguishes our case from both purely infectious cholangiopathy and from KS-related strictures amenable to endoscopic decompression.

Patients with hepatobiliary involvement often present with elevated ALP, 5–7 times the upper limit of normal and near-normal bilirubin levels. In our patient, the sclerosing cholangitis along with the KS lesion at the ampulla likely contributed to the intrahepatic cholestasis. MRCP is the preferred initial diagnostic modality for AIDS cholangiopathy and is useful in excluding alternative causes of obstructive jaundice, including gastrointestinal lymphoma. ERCP with sphincterotomy is indicated for the treatment of papillary stenosis, providing prompt relief of pain and biliary obstruction; however, it does not alter overall survival. Studies have demonstrated that sphincterotomy in the absence of papillary stenosis is not beneficial.^8^ Brush cytology or periampullary biopsy may aid in identifying the underlying causative organism. However, biliary access could not be obtained in our patient due to the presence of a KS lesion at the ampulla of Vater, preventing the acquisition of diagnostic samples (Figure 2).

**Figure 2.**
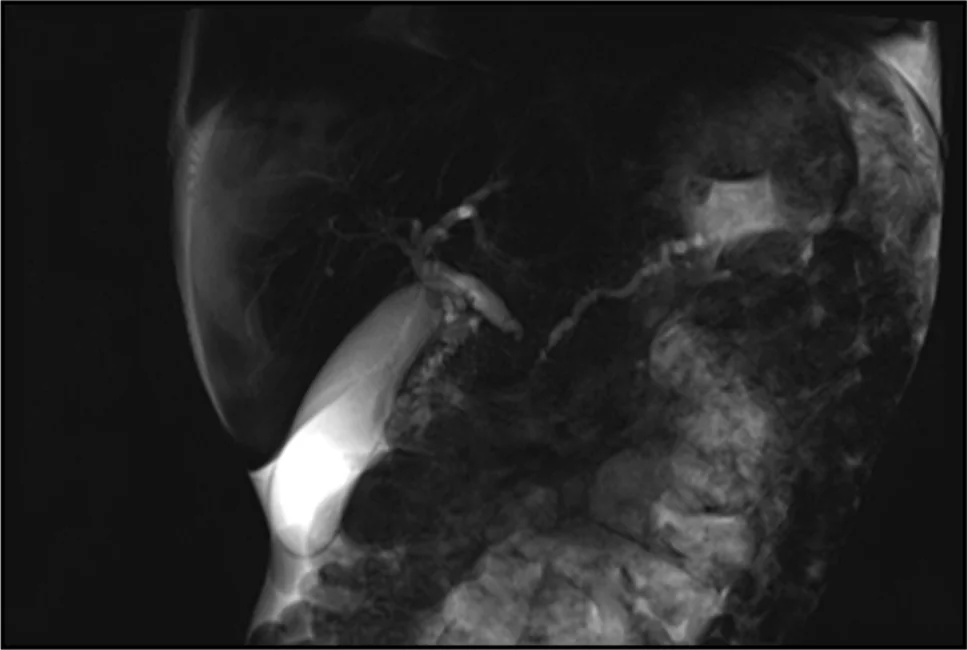
Magnetic resonance cholangiopancreatography of the abdomen showing a stricture ring at the ampulla, common bile duct measuring 0.8 cm, irregular intrahepatic bile ducts, and mildly dilated and irregular pancreatic duct with few tiny outpouchings suggestive of AIDS cholangiopathy.

In our patient, CMV-associated cholangiopathy was strongly suspected given documented CMV viremia and negative stool studies for other protozoal infections. However, ERCP revealed an obstructing, highly vascular ampullary mass, ultimately confirmed to be KS, raising the possibility of KS-related cholangiopathy. Isolated cases have been reported.^14^ A coinfection remains a plausible consideration as well. Taken together with the existing literature, this case highlights 3 distinct patterns of biliary pathology in advanced AIDS: infection-driven cholangiopathy without a structural lesion, KS-driven mechanical biliary obstruction, and coexistence of both processes, as appears to have occurred in our patient. Our interpretation is supported by the MRCP findings of irregular intrahepatic biliary and pancreatic ducts with multiple small outpouchings, features characteristic of AIDS cholangiopathy that are unlikely to be explained by mechanical obstruction from an ampullary mass alone. Conversely, the ampullary lesion identified on ERCP was independently confirmed as KS by histopathology and HHV-8 immunostaining. Accordingly, we favor concurrent AIDS cholangiopathy and ampullary KS rather than isolated KS producing secondary biliary obstruction that mimicked cholangiopathy. Nevertheless, because cholangiography and biliary brushings could not be obtained, a contributory mechanical component from the ampullary lesion cannot be completely excluded.

This case underscores the need for a high index of suspicion for both KS and AIDS cholangiopathy in patients with advanced AIDS presenting with cholestatic liver function tests. Our experience also highlights the technical challenges of biliary intervention when a highly vascular ampullary KS prevents successful cannulation, thereby limiting both diagnostic evaluation and therapeutic decompression. Consequently, management must be individualized and often requires consideration of advanced endoscopic techniques when feasible. Importantly, the patient's rapid clinical deterioration should be interpreted in the broader context of advanced, untreated HIV infection rather than attributed solely to biliary pathology. He presented with profound immunosuppression, disseminated cutaneous and hepatic KS, oropharyngeal candidiasis, and CMV viremia, reflecting multisystem disease at presentation. As demonstrated in prior reports of both AIDS cholangiopathy and KS-associated biliary disease, survival is influenced not only by successful biliary intervention but also by the severity of the underlying immunodeficiency.^11,13^ Our patient's course further emphasizes that early recognition of both infectious and neoplastic hepatobiliary complications remains essential, although prognosis ultimately depends on timely HIV treatment and the overall burden of advanced disease (Figure 3).

**Figure 3.**
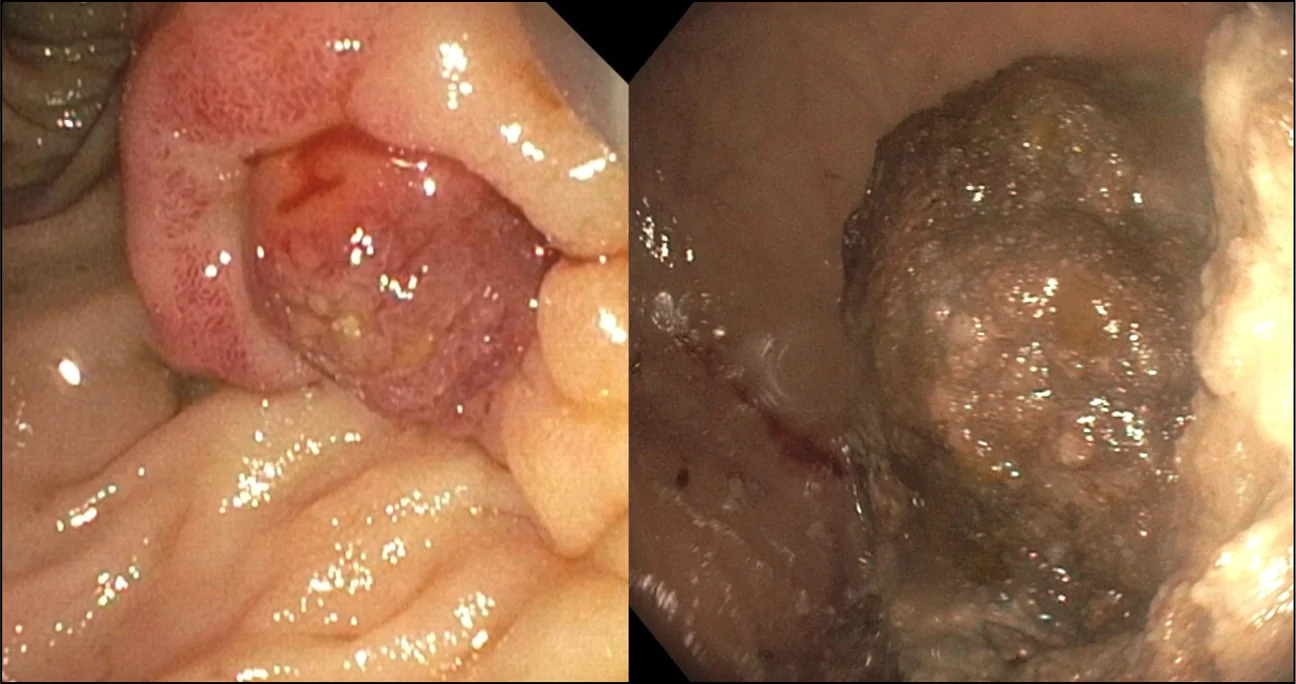
Endoscopic retrograde cholangiopancreatography images showing a large 2–3 cm umbilicated bluish vascular mass involving the ampulla, indicating Kaposi sarcoma of the ampulla of Vater.

## DISCLOSURES

Author contributions: J. Rajapandian contributed to the conception of the work, literature review, and drafting of the manuscript. CMA Hasan contributed to clinical interpretation of the case and critical revision of the manuscript. Y. Elmofti provided supervision, critical revisions, and final approval of the manuscript. All authors approved the final version and agree to be accountable for all aspects of the work. J. Rajapandian is the article guarantor.

Financial disclosure: None to report.

Previous presentation: This case was presented at the American College of Gastroenterology Annual Scientific Meeting, October 24-29, 2025; Phoenix, AZ.

Informed consent could not be obtained from the family of the deceased. All identifying information has been removed from this case report to protect patient privacy.

## ABBREVIATIONS:

AIDS: acquired immunodeficiency syndrome
ALP: alkalaine phosphatase
CBD: common bile duct
CMV: cytomegalovirus
CT: computed tomography
ERCP: endoscopic retrograde cholangiopancreatography
EUS: endoscopic ultrasound
HAART: highly active antiretroviral therapy
HHV-8: human herpesvirus 8
HIV: human immunodeficiency virus
KS: Kaposi sarcoma
MRCP: magnetic retrograde cholangiopancreatography
NAAT: nucleic acid amplification test
